# The Role of RNA Methyltransferase METTL3 in Hepatocellular Carcinoma: Results and Perspectives

**DOI:** 10.3389/fcell.2021.674919

**Published:** 2021-05-11

**Authors:** Fan Pan, Xin-Rong Lin, Li-Ping Hao, Xiao-Yuan Chu, Hai-Jun Wan, Rui Wang

**Affiliations:** ^1^Department of Medical Oncology, Affiliated Jinling Hospital, Medical School of Nanjing University, Nanjing, China; ^2^Department of Gastroenterology and Hepatology, Affiliated Jinling Hospital, Medical School of Nanjing University, Nanjing, China

**Keywords:** RNA modification, METTL3, m6A, Hepatocellular carcinoma, drug-resistance

## Abstract

Hepatocellular carcinoma (HCC) is the 6th most prevalent cancer and the 4th leading cause of cancer-related death worldwide. Mechanisms explaining the carcinogenesis of HCC are not clear yet. In recent years, rapid development of N^6^-methyladenosine (m6A) modification provides a fresh approach to disclosing this mystery. As the most prevalent mRNA modification in eukaryotes, m6A modification is capable to post-transcriptionally affect RNA splicing, stability, and translation, thus participating in a variety of biological and pathological processes including cell proliferation, apoptosis, tumor invasion and metastasis. METTL3 has been recognized as a pivotal methyltransferase and essential to the performance of m6A modification. METTL3 can regulate RNA expression in a m6A-dependent manner and contribute to the carcinogenesis, tumor progression, and drug resistance of HCC. In the present review, we are going to make a clear summary of the known roles of METTL3 in HCC, and explicitly narrate the potential mechanisms for these roles.

## Introduction

Hepatocellular carcinoma (HCC) is the sixth most prevalent cancer (4.7%) and the fourth leading cause (8.2%) of cancer-related death worldwide, with estimated 841,080 new cases and 781,631 deaths in 2018. The incidence of liver cancer presents obvious geographic heterogeneity, mostly observed in Eastern Asia and Northern Africa (Bray et al., 2018). Generally liver cancer is classified into the primary liver cancer and the secondary liver cancer. HCC, as the most significant subtype of primary liver cancer, comprises almost 75∼85% of the cases (Forner et al., 2012; Sia et al., 2017). The carcinogenesis of HCC is known as a sophisticated multistage process. Multiple risk factors have been validated to be associated with HCC, including hepatitis B virus (HBV) infection, hepatitis C virus (HCV) infection, non-alcoholic fatty liver disease (NAFLD), exposure to aflatoxin B1, alcohol intake, diabetes, and obesity (Singal and El-Serag, 2015). Despite the tremendous efforts devoted to exploring the mechanisms of hepatocarcinogenesis, few progresses have been made. Over the past decades, the rapid development of epigenetics has provided a fresh approach to disclosing the mechanisms of hepatocarcinogenesis, including DNA methylation, histone modification, chromatin remodeling, as well as RNA methylation in which N^6^-methyladenosine (m6A) modification plays an important role (Schulze et al., 2015; Villanueva et al., 2015; Xu et al., 2017; Liu et al., 2018).

**FIGURE 1:**
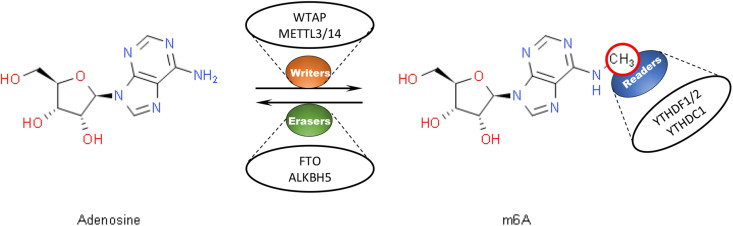
Dynamic and reversible regulation of m6A modification by regulators including “writers,” “readers,” and “erasers.” M6A “writers” serve as methyltransferases, such as WTAP and METTL3/14. M6A “readers” help recognize and bind to m6A-modified transcripts, like YTHDF1/2 and YTHDC1. M6A “erasers” have the ability for demethylation, such as FTO and ALKBH5.

N^6^-methyladenosine modification, which refers to the insertion of a methyl substituent onto the N-6 position of adenosine, is known as the most prevalent internal messenger RNA (mRNA) modification within eukaryotes (Bokar, 2005). M6A modification has been demonstrated to be capable to post-transcriptionally regulate RNA and affect RNA stability (Huang et al., 2018), splicing (Xiao et al., 2016), and translation (Lin et al., 2016). It has been proposed that m6A modification is involved in various physiological and pathological processes such as cancers (Liu et al., 2018). The conserved enrichment of m6A modification in the coding sequence (CDS) and the 3′ untranslated region (3′UTR) of mRNA has been revealed. A consensus DRACH motif (where *D* = A, G or U, *R* = A or G, *H* = A, C or U) serves as the predominant site of m6A modification (Perry et al., 1975; Csepany et al., 1990; Narayan et al., 1994; Meyer et al., 2012; Linder et al., 2015; Bayoumi and Munir, 2021). In mammalian cells, m6A modification has been found to be dynamically and reversibly regulated by several proteins which have been classified into three groups, including “erasers” with demethylation ability like FTO (Jia et al., 2011) and ALKBH5 (Tang et al., 2018), “readers” such as YTHDF1/2/3 (Li A. et al., 2017) and YTHDC1/2 (Meyer and Jaffrey, 2017; Liu J. et al., 2021) that can recognize and bind to m6A-modified transcripts, and “writers” serving as methyltransferases such as METTL3/14 and WTAP (Bokar et al., 1997; Liu et al., 2014; Figure 1). Typically, as a m6A “writer,” the METTL3-METTL14 complex has been demonstrated to be essential to the performance of m6A modification. In this complex, Methyltransferase-like 3 (METTL3), also known as MT-A70, is believed to be the only catalytic subunit (Liu et al., 2014; Ping et al., 2014).

Methyltransferase-like 3 has been observed to be substantially overexpressed and has been viewed as an adverse prognostic factor in HCC patients (Liu G. M. et al., 2020). Overexpression of METTL3 was associated with tumorigenicity and lung metastasis. *In vitro* experiments suggested that knockdown of METTL3 could lead to reduced capability of HCC cells in proliferation, migration, and colony formation (Chen et al., 2018). However, the mechanisms explaining how METTL3 contributes to HCC are not clear yet. METTL3 has been verified to function as the pivotal unit of the methyltransferase complex of m6A modification (Bokar et al., 1997). Therefore, the roles that METTL3 plays in HCC through m6A modification are worth discussion. In the present review, we are going to make an explicit summary on roles of METTL3 through m6A modification in HCC and the underlying mechanisms. Chances are that uncovering this mystery will provide us with new strategies for treatment of HCC.

## Mechanisms Underlying the Carcinogenesis and Progression of HCC

### METTL3 Suppresses SOCS2 Dependent on YTHDF2

The YTH domain family member 2 (YTHDF2) is known as a m6A “reader” that is capable to recognize and bind to m6A-modified sequences. YTHDF2 has been found associated with mRNA degradation (Du et al., 2016). Knockdown of YTHDF2 led to increased stability of target mRNAs with extended half-life of those mRNAs. Especially it is the C-terminal domain of YTHDF2 that is responsible for m6A-modified mRNA binding, while the N-terminal domain help execute the decay process (Wang et al., 2014). METTL3 was considered to be involved in YTHDF2 downstream regulation in a m6A-dependent manner. Knockdown of METTL3 led to remarkably reduced binding of YTHDF2 to its targets and thus extended the lifespan of those targets (Chen et al., 2018).

The previously reported YTHDF2 PAR-CLIP-Seq data together with transcriptome-wide m6A profiling data clearly showed the binding between the 3′ end of the suppressor of cytokine signaling 2 (*SOCS2*) transcript and YTHDF2. Consistently, knockdown of YTHDF2 significantly increased SOCS2 expression, suggesting that *SOCS2* probably served as a direct downstream target of YTHDF2 (Dominissini et al., 2012; Liu et al., 2015; Chen et al., 2018). The m6A level of *SOCS2* mRNA was strongly reduced after the knockdown of METTL3. SOCS2 expression was dramatically increased after suppressing the effect of METTL3 through the methylation inhibition. However, after mutating the adenosine bases of *SOCS2* that abolished m6A modification, METTL3 silencing could not affect the expression of SOCS2. Together, METTL3 was considered to inhibit SOCS2 expression dependent on YTHDF2 through m6A modification (Chen et al., 2018).

Clinically, low expression of SOCS2 was correlated with poor prognosis in HCC patients (Cui et al., 2016). Knockdown of SOCS2 in HCC cells substantially promoted cell proliferation and migration (Chen et al., 2018). The SOCS2, as a member of SOCS family, is a negative regulator of the cytokine-dependent Janus kinase (JAK)/signal transducer and activator of transcription (STAT) pathway. SOCS2 could inhibit the binding of STAT to its receptors and also target components of the pathways for proteosomal degradation. Enhanced JAK/STAT pathway induced by SOCS2 silencing has been indicated to play a role in cancers. Typically, STAT3 has been verified to contribute significantly to the tumorigenesis, progression, and metastasis process of HCC (Bromberg et al., 1999; Li et al., 2006; Rico-Bautista et al., 2006; Thomas et al., 2015; Xie et al., 2018).

In conclusion, overexpression of METTL3 in HCC probably facilitates the degradation process of *SOCS2* dependent on YTHDF2 and reduces its expression, thus leading to aberrant JAK/STAT pathways which is responsible for the proliferation and migration of HCC cells.

### METTL3 Inhibits RDM1

RAD52 motif-containing 1 (*RDM1*) has been verified as a target of METTL3-mediated m6A modification. Incremental expression of METTL3 decreased RDM1 expression, while it increased the m6A level of *RDM1* mRNA in HCC cell lines. Overexpression of METTL3 was also correlated with decreased expression of RDM1 in the tissues from HCC patients (Chen S. L. et al., 2020).

RAD52 motif-containing 1 has previously been regarded to be associated with tumorigenesis. Overexpression of RDM1 was observed in several cancers such as the breast cancer and lung adenocarcinoma. In those cancers, *RDM1* was regarded as an oncogene that could transcriptionally attenuate p53 expression and increase RAD51 and RAD52 level. This potential regulation of p53/RAD52/RAD51 signaling by increased RDM1 may lead to dysfunctional DNA repair pathways and the suppression of cell cycle arrest and apoptosis, thus promoting the tumor growth (Hermeking and Eick, 1994; Tong et al., 2018; Chen et al., 2019).

In HCC, it was proposed that RDM1 functioned as a tumor suppressor. Clinically low expression of RDM1 was corelated with worse differentiation, higher malignancy, and worse prognosis in HCC patients. Decreased expression of RDM1 has also been validated to improve the proliferation of HCC cells (Chen S. L. et al., 2020). RDM1 was considered to participate in DNA double-strand break (DSB) repair and recombination, which may restrain the process of carcinogenesis in HCC cells (Milne et al., 1995; Hamimes et al., 2006). Decreased expression of RDM1 was noticed to be related to stimulated calcium signaling which contributed to cancer cell survival, together with activated KRAS and RAF pathways which, as upstream of MEK/ERK pathways, enhanced cancer cell growth, survival and metabolism (Chen S. L. et al., 2020) (Asati et al., 2016; Reczek and Chandel, 2018). Overexpression of RDM1 was considered able to activate cell cycle and p53 signaling pathway (Chen S. L. et al., 2020). RDM1 could post-transcriptionally upregulate p53 expression and have a protective effect on wild-type p53, strengthening its stability and elongating its life-time. The process of DNA damage repair was predominantly facilitated by p53, serving as a crucial suppressor in HCC through unions of various DNA-damage-response (DDR) mechanisms (Staib et al., 2003; Williams and Schumacher, 2016).

In brief, RDM1 functions as a tumor suppressor in HCC by inhibiting cancer cell proliferation and promoting DNA damage repair in a p53-dependent manner. However, overexpression of METTL3 in HCC patients is able to decrease the expression of RDM1 in a m6A-dependent manner, thus promoting the survival, proliferation, and stability of HCC cells.

### METTL3 Upregulates Snail *via* YTHDF1

Recently METTL3 has been introduced to participate in the epithelial mesenchymal transition (EMT) process in HCC (Lin et al., 2019). EMT refers to the transformation of epithelial cells into mesenchymal stem cells through specific programs, providing cancer cells with the opportunities for invasion and metastasis (Chaffer et al., 2016). Knockdown of METTL3 strongly suppressed the invasion abilities and EMT process of HCC cells *in vitro*, with increased expression of E-cadherin and decreased expression of MMP2 and FN (Wong et al., 2018; Lin et al., 2019). A variety of transcription factors have been verified to be related to EMT process such as Snail, Slug, Zeb, and Twist (Puisieux et al., 2014). Typically, Snail (encoded by *SNAI1*) was suggested to be affected by METTL3 (Xu et al., 2020).

YTHDF1 is known as a m6A “reader” and able to facilitate the translation process of target mRNAs by promoting ribosome loading on those mRNAs. Knockdown of METTL3 could repress this process (Wang et al., 2015; Kapur et al., 2017). Results of m6A RIP-PCR and YTHDF1 RIP-PCR suggested that *SNAI1* was the direct target of YTHDF1 on the CDS of *SNAI1* during EMT progression. *In vitro* experiments showed that knockdown of METTL3 could reduce the expression of Snail. In tumor tissues resected from liver cancer patients, increased expression of Snail and malignant behaviors were observed, in line with elevated expression of METTL3 and YTHDF1 (Lin et al., 2019). Together, METTL3 helps increase the expression of Snail through enhanced translation of *SNAI1* mediated by YTHDF1.

Overall, overexpression of METTL3 in HCC is capable to increase the expression of Snail dependent on YTHDF1, which may promote the EMT process and provide HCC cells with opportunities for invasion and metastasis.

### METTL3 Promotes Metabolic Reprogramming

Hepatitis B virus X-interacting protein (HBXIP) has been found significantly upregulated in HCC tissues and HCC cell lines (Melegari et al., 1998). Overexpression of HBXIP has been demonstrated to be associated with poor prognosis of HCC patients (Zheng et al., 2019). Several mechanisms have been proposed to account for HBXIP’s oncogenic roles. Among these mechanisms, metabolism reprogramming has been validated to be associated with METTL3 in HCC (Yang N. et al., 2020; Xiu et al., 2021). Liver cancer cells is metabolically characterized by the Warburg effect (or aerobic glycolysis), with enhanced glycolysis and increased level of lactic acid (Koppenol et al., 2011; Vaupel et al., 2019). METTL3, which was positively regulated by HBXIP in HCC, has been verified to be involved in metabolic reprogramming. According to the gene set enrichment analysis, expression of METTL3 was found positively correlated with the expression of genes involved in glycolysis such as glucose transporter member 1 (*SLC2A1*), hexokinase 2 (*HK2*), and pyruvate kinase (*PKM*), while it was negatively correlated with the expression of gluconeogenesis-related genes like glucose-6-phosphatase catalytic subunit (*G6PC*), pyruvate carboxylase (*PC*), and Fructose-1,6-bisphosphatase (*FBP1*) (Lin Y. et al., 2020). It seems that overexpression of METTL3 could promote the glycolysis process and inhibit the gluconeogenesis process. Knockdown of METTL3 was noticed able to repress glycolysis process and activate TCA cycle in HCC cells, with suppressed capability for cell aggression. Further experiments demonstrated that HBXIP’s role in metabolic reprogramming in HCC was dependent on METTL3 (Yang N. et al., 2020).

Hypoxia-Inducible Factor 1α (HIF-1α), associated with the genesis and development of tumors, has been demonstrated to promote the glycolysis process and facilitate the carcinogenesis in HCC (Gwak et al., 2005; Chiavarina et al., 2012; Lin and Wu, 2015). Overexpression of HIF-1α was associated with promoted metabolic reprogramming. METTL3 has been verified to regulate HIF-1α expression in a m6A-dependent manner. Results of MeRIP-qPCR demonstrated that METTL3 could increase the m6A level of *HIF-1α* mRNA. Knockdown of METTL3 strongly reduced the expression of HIF-1α (Yang N. et al., 2020).

Another strategy that accounted for how METTL3 participated in metabolic reprogramming was proposed. The mammalian target of rapamycin complex 1 (mTORC1) signaling was introduced as a crucial signaling that was involved in cell metabolism. MTORC1 signaling has been demonstrated to promote the glycolysis process (Laplante and Sabatini, 2012; Tian et al., 2019). Expressions of genes that encoded the enzymes of almost every step of glycolysis were found upregulated in response to activation of mTORC1, such as *ALDOA*, *HK1/2*, and *SLC2A1/GLUT1* (Düvel et al., 2010). It was proposed that METTL3 potentially targeted the mTORC1 to regulate the glycolysis process in HCC. Impaired mTORC1 activity was observed after knockdown of METTL3, with reduced phosphorylation of S6K1 and 4EBP1 which were both substrates of mTORC1 (Burnett et al., 1998; Lin Y. et al., 2020). Additional silencing of METTL3 was unable to further decrease the phosphorylation level of mTORC1 and glycolysis activity in Rapamycin-treated HCC cells, suggesting the regulation of METTL3 on mTORC1. How mTORC1 signaling affected the glycolysis process has been researched. A c-Myc-LDHA axis was proposed to be a downstream target of mTORC1 and contribute to the abnormal glycolysis (Dang et al., 2009; Zhao et al., 2016). In addition, HIF-1α could function as another downstream effector of mTORC1 and participate in this process (Semenza et al., 1994; de la Cruz López et al., 2019).

In summary, overexpression of METTL3 in HCC could owe to the increased expression of HBXIP and be responsible for metabolic reprogramming and the proliferation, migration and invasion of HCC cells. *HIF-1α* is a potential target of METTL3-mediated m6A modification and is involved in metabolic reprogramming. Enhanced mTORC1 signaling is also closely related to METTL3 overexpression and associated with aberrant glycolysis process.

### METTL3 Increases Lipogenesis *via* Upregulation of LINC00958

In addition to mRNAs, METTL3 has been associated with long non-coding RNAs (lncRNAs) in cancers including HCC (Zuo et al., 2020; Liu G. M. et al., 2021; Qu et al., 2021; Rong et al., 2021). lncRNAs, which comprises about 4–9% of total RNAs, refers to RNAs with limited or no protein-coding potential and possesses transcript sequence of more than 200 nt in length. LncRNAs are believed to execute the function of regulating gene expression and are involved in a variety of biological and pathological processes including cancers. Some lncRNAs have been demonstrated to be involved in carcinogenesis of HCC (Esteller, 2011; Gong et al., 2017; Ji et al., 2019; Ye et al., 2020). Here we take lncRNA *LINC00958* as an example and demonstrate the effects on LINC00958 by METTL3, together with the detailed mechanisms explaining how this process affects HCC.

Overexpression of LINC00958 was observed in HCC and correlated with malignant behaviors of HCC cells and poor prognosis in HCC patients. Overexpression of LINC00958 was likely to promote cell growth, proliferation, migration and invasion in HCC cells. Clinically LINC00958 expression was associated with tumor size, tumor differentiation, microvascular invasion, and TNM stage. M6A RIP-qPCR analysis demonstrated that m6A level on *LINC00958* was increased appreciably in HCC cells. Knock down of METTL3 reduced the m6A level of *LINC00958* which probably decreased the stability of *LINC00958* transcript and reduced its expression, suggesting that METTL3 in HCC may positively regulate LINC00958 expression (Liu G. M. et al., 2020; Zuo et al., 2020).

A LINC00958/miR-3619-5p/HDGF axis was proposed. Hepatoma-derived growth factor (HDGF) was regarded as an independent prognostic factor in liver cancer and was significantly upregulated in HCC (Zhou et al., 2010). Aberrant lipogenesis process *via* HDGF may account for its oncogenic characteristics. HDGF served as a coactivator of the sterol regulatory element binding protein-1 (SREBP-1) to participate in transcriptional activation of lipogenic enzymes associated with fatty acid, triglyceride, and cholesterol synthesis in HCC (Goldstein et al., 2006; Min et al., 2018). HDGF was a direct target of miR-3619-5p. Expression of HDGF has been demonstrated to be negatively regulated by miR-3619-5p (Zuo et al., 2020). However, as a sponge of miR-3619-5p, LINC00958 was regarded to be involved in abnormal lipogenesis process. Competitively binding to miR-3619-5p prevented the interaction between miR-3619-5p and HDGF, resulting in overexpression of HDGF (Peng et al., 2017). With the overexpression of LINC00958, HCC cells exhibited increased cellular levels of cholesterol and triglyceride, suggesting that LINC00958 positively regulated lipogenesis process.

In a word, overexpression of LINC00958 may be ascribed to METTL3-mediated m6A modification. A LINC00958/miR-3619-5P/HDGF axis was proposed to explain how LINC00958 affects lipogenesis and contributes to HCC.

## Mechanisms Accounting for Drug-Resistance of HCC

### METTL3 Depletion Contributes to Sorafenib Resistance *via* FOXO3

Apart from promoting the carcinogenesis and progression of HCC, METTL3 has been related to the resistance of anti-HCC drugs, such as the sorafenib resistance. Clinically, METTL3 silencing was found to noticeably enhance sorafenib resistance in HCC patients (Lin Z. et al., 2020).

Sorafenib has been known as a multi-target oral drug for treatment of tumors. It had dual anti-tumor effects aiming at both tumor cell growth and tumor angiogenesis. Sorafenib functioned as a multi-kinase inhibitor. It was capable to suppress tumor cell proliferation by inhibiting RAF/MEK/ERK pathway, and repress the angiogenesis process through impeding vascular endothelial growth factor receptor (VEGFR) and platelet-derived growth factor receptor (PDGFR). Sorafenib was the only FDA-approved drug for first-line treatment of advanced HCC and has been validated to prolong the overall survival (OS) of those patients (Cheng et al., 2009; Iyer et al., 2010; Brunetti et al., 2019). However, both primary and acquired resistance to sorafenib have been reported during clinical application.

Hypoxia was observed in HCC resulting from inadequate perfusion and diffusion in tumor tissues, with obvious damaged oxygenation status (Vaupel et al., 2007). Tumor tissues obviously showed higher expression of HIF-1α comparing to adjacent normal tissues. In this situation, reduced expression of METTL3 was noticed in sorafenib-resistant HCC. Catalytic mutant METTL3 did not sensitize METTL3-knockdown HCC cells for sorafenib treatment, suggesting METTL3’s m6A-dependent roles as a methyltransferase. Increased level of autophagosomes and LC3 were also observed in sorafenib-resistant HCC, and could be reversed by overexpression of wild-type METTL3, indicating that autophagy process may be associated with METTL3 and responsible for sorafenib-resistance (Lin Z. et al., 2020). The autophagy process has been considered to participate in multidrug resistance in chemotherapy of cancer (Li Y. J. et al., 2017). FOXO3, as has been elucidated to be associated with autophagy, was introduced to further explain the mechanisms. Knock down of FOXO3 facilitated the transcription of autophagy-related genes and was related to enhanced autophagy. Knock down of METTL3 decreased both mRNA and protein level of *FOXO3*, which could be reversed by wild-type METTL3 other than catalytic mutant METTL3. RNA m6A-Seq suggested that *FOXO3* was probably the direct target of YTHDF1 which is a m6A “reader” and promotes the translation of its targets. The m6A site was located at the 3′UTR region (Wang et al., 2015; Fitzwalter and Thorburn, 2018; Lin Z. et al., 2020). In summary, Knockdown of METTL3 probably reversed the increasing transcription efficiency of *FOXO3* mediated by YTHDF1 and brought about decreased FOXO3 expression, thus facilitating transcription of genes related to autophagy including *ATG3/5/7/12*, *ATG16L1*, and *MAP1LC3B* in HCC, ultimately leading to resistance of sorafenib (Lin Z. et al., 2020).

Briefly, under the circumstance of hypoxic microenvironment within tumors, METTL3 depletion has been discovered to substantially contribute to the acquired sorafenib resistance in HCC *via* FOXO3-mediated autophagy. Thus, METTL3 is a promising target to reverse sorafenib resistance in chemotherapy of HCC patients.

## Discussion

**FIGURE 2:**
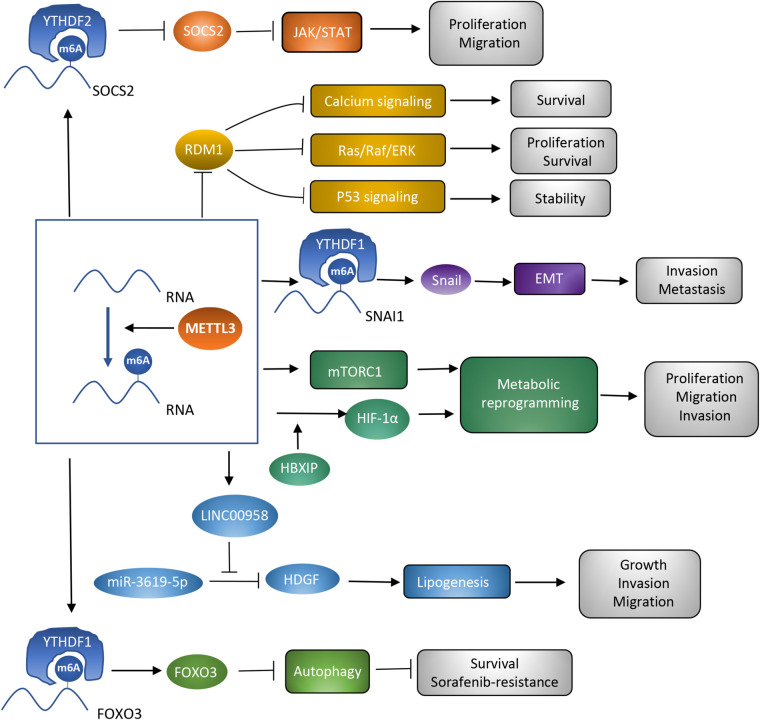
Roles of METTL3 as a methyltransferase in HCC. Different targets of METTL3 are associated with diverse signaling pathways and biological processes, which contribute to the different influences of METTL3 on HCC.

In the last decades, m6A modification has been researched to clarify the potential mechanisms accounting for various kinds of cancers (Sun et al., 2019). METTL3, as a critical subunit of the METTL3-METTL14 methyltransferase complex, has been validated to contribute to the process of cancer. The present review focuses on hepatocellular carcinoma, and demonstrates several potential targets of METTL3 and the way METTL3 contributes to HCC in a m6A-dependent manner. METTL3 has been verified to be involved in proliferation, invasion, and metastasis of HCC cells, as well as the glycolysis and lipogenesis processes, promoting the carcinogenesis and progression of HCC (Chen et al., 2018; Chen S. L. et al., 2020; Lin Z. et al., 2020; Xu et al., 2020; Yang N. et al., 2020; Zuo et al., 2020; Table 1). Therefore, METTL3 may function as a potential target of anti-HCC treatment. However, METTL3 silencing has also been noticed to be correlated with sorafenib resistance in chemotherapy of advanced HCC patients. Mechanisms explaining how METTL3 serves as a double-edged sword in HCC deserve discussion. Overexpression of METTL3 was correlated with activation of JAK/STAT and Ras/Raf/ERK pathways, repression of p53 signaling pathway, and enhancement of metabolic reprogramming and the EMT process. Regulation by METTL3 on these signaling pathways and biological processes resulted in carcinogenesis and progression of HCC. However, the sorafenib resistance owed to the enhanced autophagy process which acted as a protective mechanism in cancer and could be induced by METTL3 silencing. Different targets of METTL3 accounted for its distinct influences (Figure 2). Clearly narrating these mechanisms helps support the clinical application of METTL3 in treatment of HCC. Apart from the mechanisms that have already been discussed in this review, some other potential targets of METTL3 may account for the carcinogenesis, progression, and drug resistance process in HCC. More efforts are required to further disclose METTL3’s role in HCC.

**TABLE 1:** Roles of METTL3 in HCC.

**Target RNAs**	**Signaling pathways**	**Biological processes**	**Cellular function**	**References**
*SOCS2*	JAK/STAT signaling		Cell proliferation, migration	Chen et al., 2018
*RDM1*	Calcium signaling, Ras/Raf/ERK signaling, p53 signaling		Cell survival, proliferation, stability	Chen S. L. et al., 2020
*SNAI1*		EMT	Cell invasion and metastasis	Xu et al., 2020
	mTORC1 signaling	Metabolic reprogramming	Cell proliferation, migration, invasion	Yang N. et al., 2020
*HIF-1α*		Metabolic reprogramming	Cell proliferation, migration, invasion	Lin Y. et al., 2020
*LINC00958*		lipogenesis	Cell growth, invasion, migration	Zuo et al., 2020
*FOXO3*		autophagy	Cell survival, sorafenib resistance	Lin Z. et al., 2020

*Some potential targets of METTL3 have already been researched and discovered to be associated with the carcinogenesis, progression, and sorafenib-resistance process of HCC.*

It should be noticed that m6A sequencing has been applied to discuss the interaction between METTL3 and its targets. However, the validity of m6Aseq is still unclear and needs extensive validation. LncRNAs have been associated with m6A modification in some cancers (Zuo et al., 2020; Liu G. M. et al., 2021; Qu et al., 2021; Rong et al., 2021). In the present review, we talked about *LINC00958* which was positively correlated with METTL3 and was involved in aberrant lipogenesis in HCC (Zuo et al., 2020). Nevertheless, the detailed mechanisms explaining how METTL3 regulates LINC00958 are lacking. Extensive researches are required to further explain the regulation of METTL3 on lncRNAs. Some researches individually focused on METTL3 and its effects on HCC. Considering that METTL3 and METTL14 collectively form the methyltransferase complex, it is necessary to discuss the interaction between METTL3 and METTL14. In addition to m6A “writers,” some other regulators of m6A modification including YTHDC2, ALKBH5 and FTO have been associated with HCC (Li J. et al., 2019; Chen Y. et al., 2020; Liu J. et al., 2021). It is necessary to disclose their roles and the relevant mechanisms, which could promote their applications in clinical practice of HCC treatment.

Interestingly, opposite regulatory roles of METTL3 and METTL14 were observed in some cancers including HCC (Ma et al., 2017; Chen et al., 2018; Li T. et al., 2019; Yang X. et al., 2020). METTL14 was reported to share almost 56% binding sites with METTL3 (Liu et al., 2014). Functionally, METTL14 was regarded to structurally stabilize METTL3 conformation and help substrate recognition (Wang et al., 2016) Decreased expression of METTL14 was seen in HCC and was correlated with migration, invasion and EMT of HCC cells (Shi et al., 2020). Clinically, HCC patients with lower expression of METTL14 showed poorer prognosis, with lower OS rate. According to a multi-omics analysis, most of the mRNAs, signaling pathways and biological processes were differently regulated after knock down of METTL3 and METTL14, potentially explaining the distinct roles of METTL3 and METTL14 in HCC (Liu X. et al., 2020). More researches are required to clarify the disparate effects of METTL3 and METTL14 on HCC and the relevant mechanisms.

## Author Contributions

RW conceptualized the review. FP were the major contributors in writing the manuscript. X-RL and L-PH designed the figures. X-YC and H-JW critically reviewed and edited the manuscript. All the authors read and approved the final manuscript.

## Conflict of Interest

The authors declare that the research was conducted in the absence of any commercial or financial relationships that could be construed as a potential conflict of interest.
